# Soliton formation and spectral translation into visible on CMOS-compatible 4H-silicon-carbide-on-insulator platform

**DOI:** 10.1038/s41377-022-01042-w

**Published:** 2022-12-07

**Authors:** Chengli Wang, Jin Li, Ailun Yi, Zhiwei Fang, Liping Zhou, Zhe Wang, Rui Niu, Yang Chen, Jiaxiang Zhang, Ya Cheng, Junqiu Liu, Chun-Hua Dong, Xin Ou

**Affiliations:** 1 State Key Laboratory of Functional Materials for Informatics, Shanghai Institute of Microsystem and Information Technology, Chinese Academy of Sciences, 200050 Shanghai, China; 2 The Center of Materials Science and Optoelectronics Engineering, University of Chinese Academy of Sciences, 100049 Beijing, China; 3 CAS Key Laboratory of Quantum Information, University of Science and Technology of China, 230026 Hefei, China; 4 CAS Center for Excellence in Quantum Information and Quantum Physics, University of Science and Technology of China, 230026 Hefei, China; 5 The Extreme Optoelectromechanics Laboratory (XXL), School of Physics and Electronic Science, East China Normal University, 200241 Shanghai, China; 6 State Key Laboratory of High Field Laser Physics and CAS Center for Excellence in Ultra-intense Laser Science, Shanghai Institute of Optics and Fine Mechanics, Chinese Academy of Sciences, 201800 Shanghai, China; 7 International Quantum Academy, 518048 Shenzhen, China; 8 Hefei National Laboratory, University of Science and Technology of China, Hefei, 230026 China

**Keywords:** Nonlinear optics, Microresonators

## Abstract

Recent advancements in integrated soliton microcombs open the route to a wide range of chip-based communication, sensing, and metrology applications. The technology translation from laboratory demonstrations to real-world applications requires the fabrication process of photonics chips to be fully CMOS-compatible, such that the manufacturing can take advantage of the ongoing evolution of semiconductor technology at reduced cost and with high volume. Silicon nitride has become the leading CMOS platform for integrated soliton devices, however, it is an insulator and lacks intrinsic second-order nonlinearity for electro-optic modulation. Other materials have emerged such as AlN, LiNbO_3_, AlGaAs and GaP that exhibit simultaneous second- and third-order nonlinearities. Here, we show that silicon carbide (SiC) -- already commercially deployed in nearly ubiquitous electrical power devices such as RF electronics, MOSFET, and MEMS due to its wide bandgap properties, excellent mechanical properties, piezoelectricity and chemical inertia -- is a new competitive CMOS-compatible platform for nonlinear photonics. High-quality-factor microresonators (*Q* = 4 × 10^6^) are fabricated on 4H-SiC-on-insulator thin films, where a single soliton microcomb is generated. In addition, we observe wide spectral translation of chaotic microcombs from near-infrared to visible due to the second-order nonlinearity of SiC. Our work highlights the prospects of SiC for future low-loss integrated nonlinear and quantum photonics that could harness electro-opto-mechanical interactions on a monolithic platform.

## Introduction

Dissipative Kerr solitons (DKS) are self-organized stable optical pulses generated under the interaction between anomalous group velocity dispersion (GVD) and Kerr nonlinearity in optical resonators^1,2^. First demonstrated in fibers^1,3^, recent interest and effort have been made on DKS generation in integrated microresonators^4–6^. Microresonator-based solitons, as sources for low noise, broadband optical frequency combs, exhibit various unique properties such as repetition rates in the microwave to terahertz region^7–9^ and chip-scale footprints^10,11^. Soliton microcombs have been already used for a myriad of applications including communications^12^, spectroscopy^13,14^, and time-keeping^15^.

Future deployment of chip-based soliton microcombs in optical communication systems and datacenters requires the realization of multiple optoelectronic functions on the same chip. This naturally raises the necessity to investigate different material platforms^16^ that feature low optical loss, large refractive index, high Kerr nonlinearity, tailorable dispersion, and ideally compatibility with existing CMOS fabrication technology. In the last decade, we have witnessed the generation of soliton microcombs based on various material platforms, including silica^9,17^, Si_3_N_4_^18–24^, Hydex^8,25^, AlN^26,27^, LiNbO_3_^28,29^, AlGaAs^30,31^, and GaP^32^. Among these, AlN, LiNbO_3_, AlGaAs, and GaP feature simultaneous intrinsic second- and third-order nonlinearities, thus are particularly promising for the generation and on-chip actuation of soliton microcombs. However, high-quality AlN thin films can only be epitaxially grown on the sapphire substrates^26^. While LiNbO_3_ has a strong Pockels coefficient and is widely used for electro-optic modulators, the current fabrication process of monolithic LiNbO_3_ photonic integrated circuits is not yet compatible with CMOS foundries developed for silicon photonics. In contrast, integrated photonics based on AlGaAs and GaP can directly benefit from the existing fabrication facility developed for III-V semiconductors. However, these materials have not yet demonstrated coherent soliton generation at room temperature due to the exaggerated thermal-optic effects and absorption losses^33,34^.

Recently, silicon carbide (SiC), especially its 4H polytype, has emerged as another promising CMOS-compatible platform for electro-optic and integrated nonlinear photonics^35–37^. Compared with Si_3_N_4_, SiC features a larger refractive index (*n* = 2.6) and a strong nonlinear refractive index of *n*_2_ = 8.6 × 10^−18^ m^2^W^−1^ ^38^. In addition, the non-centrosymmetric lattice structures of SiC endow an intrinsic second-order nonlinearity that is essential for second-harmonic generation and potentially self-referencing of optical frequency combs. Meanwhile, SiC also exhibits Pockels effects that can be used for fast electro-optic manipulation of the microcomb on the same chip^39^. Moreover, as SiC has already been widely used in micro-electro-mechanical systems (MEMS) due to its excellent mechanical properties, piezoelectricity and chemical inertia^40^, SiC integrated photonics can naturally combine with MEMS technology and thus create hybrid systems harnessing electro-opto-mechanical interactions^41^. Furthermore, 4H-SiC hosts many optical semiconductor spin-qubits for quantum technology^42,43^. Finally, it is worth to stress that, due to its excellent electrical properties and ubiquitous applications in electrical power devices such as RF electronics^44^ and metal-oxide-semiconductor field-effect transistor (MOSFET)^45^, the scale and maturity of the well-established SiC manufacturing technology can directly benefit its use in integrated photonics.

All these unique properties highlight that SiC is promising for integrated nonlinear photonics, in particular soliton microcombs. Recent breakthroughs have shown thin-film 4H-SiC-on-insulator (4H-SiCOI)^36,37^ used for soliton microcomb generation at 4 K cryogenic temperature^46^, or octave spanning modulation-instability microcombs under high input power (120 mW)^47^. Despite these advances, the strong χ^2^ nonlinearity of SiC remains unexplored. Meanwhile, although the methods to generate soliton microcombs at cryogenic temperature have been well developed and can significantly reduce the thermal-optic effect^33,46,48^, the overall setup is relatively complicated, which is undesired for field-deployable applications.

In this work, we generate soliton microcombs at room temperature in the low-loss 4H-SiCOI integrated platform. We obtain different soliton states, including soliton crystals and the single soliton. We further observe wideband spectral translation of chaotic microcombs from near-infrared (IR) to visible wavelength via simultaneous χ^2^ and χ^3^ nonlinearities in our high-quality (Q) 4H-SiCOI microresonators, in contrast to other CMOS-compatible materials such as Si_3_N_4_ where the χ^2^ nonlinearity is absent.

## Results

### SiC microresonator characterization for comb generation

The fabrication process of integrated photonic microresonators based on a 4H-SiCOI wafer starts with bonding SiC to an oxidized Si handle wafer at room temperature. The bonded SiC layer is mechanically grinded, followed by chemical-mechanical polishing (CMP) of the bonded SiC layer to a thickness of several micrometers. Afterwards, the SiC layer is dry-etched to the target thickness, usually <1 µm. The quality of the final SiC thin film can be the same as that of virgin SiC wafers, thus allowing us to fabricate high-*Q* factor SiC microresonators thereon. The SiC microdisk resonator is fabricated using a femtosecond laser-assisted CMP (see ‘Method’)^36^. This process enables an ultrasmooth sidewall of the microresonator, vital for achieving high microresonator *Q* factors. Meanwhile, the bonding is carried out at room temperature, and subsequent high-temperature annealing is not required, the entire SiC process as well as used materials are fully CMOS-compatible.

**Fig. 1:**
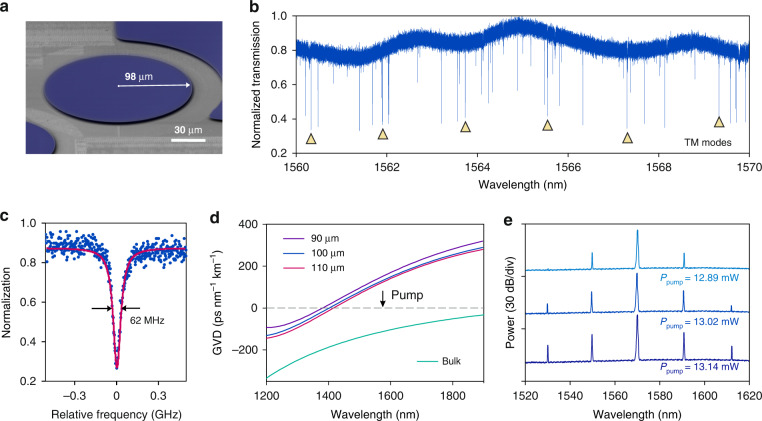
Characterization of 4H-SiC integrated photonic microresonators. **a** False-colored scanning electron microscopy (SEM) image of a 4H-SiC microdisk resonator with a radius of 98 µm. The SiC is blue-colored. **b** Normalized power transmission spectrum of an over-coupled microresonator with the transverse-magnetic (TM) polarization. The fundamental TM mode family is identified and marked by triangular symbols. Other resonances belong to higher-order TM modes of the microresonator. **c** A resonance of the fundamental TM mode with a loaded linewidth of 62 MHz extracted from the Lorentzian fit (red line), corresponding to a loaded *Q* factor of 3.1 × 10^6^. **d** Numerical simulations of group-velocity dispersions (GVD) of 800 nm thick SiC microdisks with different radii. **e** Optical spectrum of the initially parametric oscillation with different input power

Figure 1a shows a false-colored scanning electron micrograph (SEM) image of the fabricated SiC microresonator. A tapered optical fiber is used to couple light into the microresonator. The over, critical, and under coupling states can be controlled by adjusting the gap between the fiber and the microresonator (see Supplementary Note S1). Although the critical coupling has higher efficiency, maintaining the critical coupling state is sensitive to the vibration of the system, and optomechanical vibration^49^ is observed when a high pump power is used to generate solitons (see Supplementary Note S2). Here, instead, we operate the system in the over coupling state^50^, where the tapered fiber is attached to the microdisk edge (see Supplementary Note S1). Figure 1b depicts the transmission spectrum for the TM polarization in the over-coupled condition. Multiple TM mode families are observed, and the fundamental TM mode family is identified (see Supplementary Note S1). The SiC microresonator has a free space range (FSR) of 208 GHz. Figure 1c shows a measured resonance linewidth which corresponds to a loaded *Q* factor of 3.1 × 10^6^ and an intrinsic *Q* factor of 4 × 10^6^. It is worthy to mention that, such a high intrinsic *Q* factor in SiC is on par with the state of the art of thin-film LiNbO_3_, is only achieved in Si3N4 film annealed above 1100 °C^20^.

To generate microcombs in a Kerr-nonlinear microresonator, anomalous GVD is required to counteract cross- and self-phase modulation^11,51^. Owing to the strong normal materials GVD of SiC, finite-element simulations reveal that the microresonator thickness less than 1 µm is required to obtain anomalous GVD at telecommunication bands for the fundamental TM mode. Figure 1d plots the simulated GVD of the fundamental TM mode of SiC microresonators with 800 nm thickness but different radii. In our work, we use the SiC microdisk with 98 µm radius and 800 nm thickness. Notably, the fundamental TE mode exhibits anomalous dispersion. This is the reason why the TM mode is dispersion-engineered and used for soliton microcomb generation. Soliton microcombs are initially seeded through optical parametric oscillation, whose typical spectra are shown in Fig. 1e. A series of widely spaced sidebands are generated with input power of around 13 mW. The output power of the primary sidebands as a function of the input power is plotted in Supplementary Note S3, revealing a threshold power of 12.9 mW and a calculated nonlinear refractive index of *n*_*2*_ = 6 × 10^−19^ m^2^ W^−1^ (see the Supplementary Note S3 for more details). This value is consistent with previously reported results^38,52^.

**Fig. 2:**
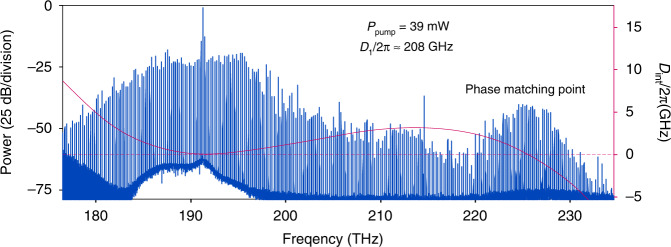
Generated frequency comb spectrum with the pump power of 39 mW. The pink solid line plotted the integrated dispersion *D*_*int*_ = 0 from FEM simulation according the microresonator geometry. The phase matching point revealed from the simulations, where *D*_*int*_ = 0, agrees with the spectral position where enhanced comb line power is observed

As depicted in Fig. 2, a broadband frequency comb is generated upon further increasing the pump power and adjusting the detuning. The comb spectrum spans over 350 nm with a native frequency spacing (single FSR). This sharp feature around 220 THz is due to zero integrated dispersion where the higher-order dispersion term^53^ counteract the GVD and broadband phase matching is achieved. The integrated dispersion *D*_*int*_ = *ω(µ) − (ω*_*0*_ + *µD*_*1*_*)* calculated from FEM simulation based on the actual device geometry is shown in the pink solid line in Fig. 2, where the zero-dispersion point *D*_*int*_ = 0 is achieved around 220 THz, in agreement with the measured spectrum.

### Soliton formation in SiC microresonators

Bright dissipative soliton formation in optical microresonators relies on the double balances between the cavity dispersion and Kerr nonlinearity, along with parametric gain and cavity loss^2^. The mode family aimed for soliton formation should exhibit anomalous GVD and also a minimal distortion of the dispersion profile caused by avoided mode crossings^2,54^. The first of these requirements has been satisfied in our SiC microresonators. However, the second requirement is more challenging in this work. As shown in Fig. 1b, due to the high refractive index of SiC and the over coupling state of the experiment, several mode families appear in the spectrum, causing strong avoided mode crossing^55^ and local dispersion distortion that hinder soliton formation^54^. In addition, generating and maintaining soliton that resides on the red-detuned side of the resonance often suffer from strong thermo-optic instability, especially in microresonators featuring positive and large thermo-optic coefficient *dn/dT*. The thermo-optic coefficient of our SiC microresonator is quantitatively measured to be 4.67 × 10^−5^ K^−1^ (see Supplementary Note S4), seven times larger than that of silica (8 × 10^−6^ K^−1^), which creates difficulty to access solitons using the common fast frequency scanning method^2,9^.

**Fig. 3:**
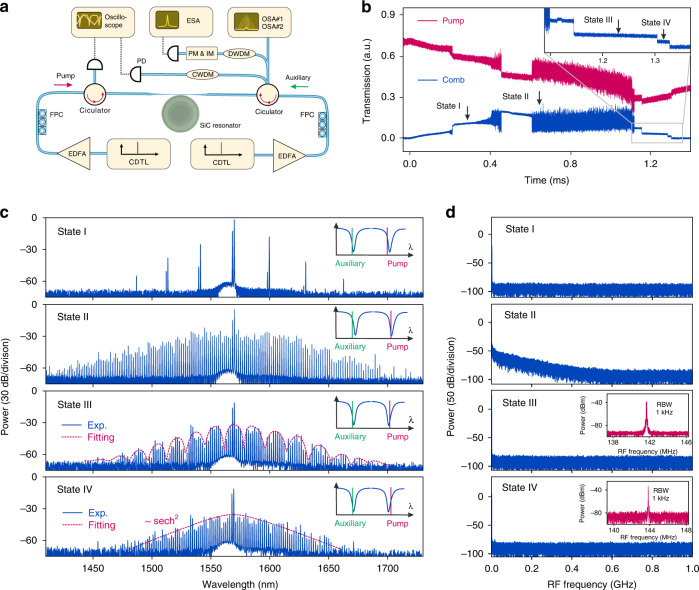
Soliton formation in the 4H-SiC microresonator. **a** Experimental setup. CDTL continuously diode tunable laser, EDFA erbium-doped fiber amplifier, FPC fiber polarization controller, PD photodetector, DWDM dense wavelength division multiplexer, CWDM coarse wavelength division multiplexer, PM phase modulator, IM intensity modulator, ESA electrical spectrum analyzer, OSA optical spectrum analyzer. **b** The microresonator transmission spectrum of the pump (red) and the generated comb power (blue) when the pump laser frequency is scanned from the blue-detuned to the red-detuned of the pump resonance. The step features observed in both traces signal soliton formation. **c** The optical power spectra recorded at four different positions marked in **b**. The dashed red lines in states III and IV are fitted soliton spectral envelope. The insets illustrate the detuning positions of the pump (red) and auxiliary (green) lasers to their respective resonances. **d** The RF amplitude noise spectra corresponding to the four different states. The insets show the RF beatnote with limited resolution-bandwidth of 1 kHz

**Fig. 4:**
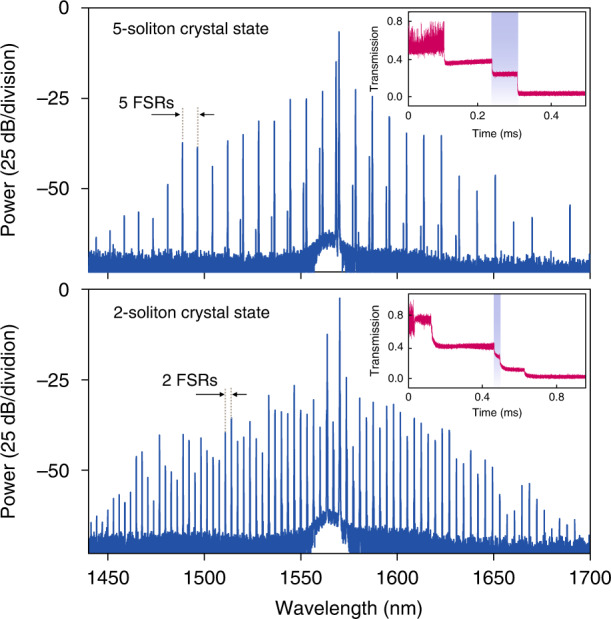
Optical spectra of the soliton crystal states. The insets show the transmission traces of the comb power, the marked region is the corresponding soliton state when the pump laser is adjusted to this stage. Top, 5-soliton crystal state; Bottom, 2-soliton crystal state

Previously, the access to the soliton regime required to operate the experiment in the 4K cryogenic temperature^46^ to suppress the thermo-optic effect. Here, in contrast, we utilized an auxiliary laser^56–58^ in addition to the pump laser to suppress the thermo-optic effect in SiC at room temperature. Figure 3(a) shows the experimental setup. The pump and auxiliary lasers from two tunable external-cavity diode lasers are amplified with two EDFAs, and are coupled into the SiC microresonator in opposite directions. The signal is collected through the same tapered fiber, and its spectral and noise characteristics are analyzed. In our experiment, the pump and auxiliary lasers are firstly stabilized in the blued-detuned side of two different resonances, respectively. Then, the pump laser is swept over the pump resonance. The transmitted pump laser, as well as the generated microcomb power, are recorded and depicted in Fig. 3b. Typical step features signaling the transition from the chaotic comb states to coherent soliton states are observed, from which the formation of solitons can be inferred. The corresponding optical comb spectra and RF amplitude noise spectra with different laser frequency detuning are plotted in Fig. 3c, d, including the initial primary comb (state I), modulation instability comb (state II), a multi-soliton comb (state III), and a single soliton comb (state IV). The insets in Fig. 3c illustrate the frequency detuning of the pump and auxiliary lasers relative to their coupled resonances. The presence of the blue-detuned auxiliary laser can compensate the thermal recoil during the transition from the chaotic regime to a soliton state and thus favor the formation of solitons at room temperature^56^. The spectral envelope of the multi-soliton comb (state III) features a sinusoidal modulation with a period of 20 nm due to interfering Fourier components between the solitons circulating in the microresonator. From the fitting of the comb spectral envelope (see ‘Methods section’), the soliton number is resolved and their relative angle is calculated. As depicted in state III in Fig. 3c, the fitting curve matches the spectral envelope, corresponding to two solitons circulating in the microresonator with a relative angle of 35.1°. Further increasing the pump laser detuning leads to transition to state IV, corresponding to the soliton stage in Fig. 3b. The spectral envelope is fitted with a sech^2^ shape of the single soliton envelope. The RF spectra also confirm low-noise soliton formation, and the beat notes of two adjacent comb lines exhibit a signal-to-noise ratio exceeding 50 dB (resolution bandwidth is 1 kHz). The irregular spectral envelope found in state IV is likely caused by severe avoided mode crossing^5,54,59^. A soliton crystal^17,59^ state consisting of temporally ordered ensembles of copropagating solitons is observed. Figure 4 shows two optical spectral of soliton crystals with 5- and 2-FSR line spacings, corresponding to 5 and 2 identical soliton crystals, respectively. The transmitted comb power traces plotted in the inserts confirm soliton formation. Our observation agrees with previous works that demonstrated soliton crystals formation due to strong avoid mode crossing^8,17,58,60^.

### Comb spectral translation from near-infrared to visible

Frequency combs in the visible wavelength are key for applications such as optical clocks, biomedical imaging and spectroscopy^61–63^. However, generating microcombs in the visible wavelength is challenging, especially in CMOS-foundry-based materials. The main obstacle is the strong normal material GVD as well as the degraded *Q* factors at short wavelengths. Although recent advances have shown that the visible microcombs can be realized using higher-order spatial mode engineering^64^ or specific cavity geometries^65^, the comb bandwidth is still very limited. An alternative is to spectrally translate microcombs from near-IR to visible using microresonators that simultaneously exhibit strong χ^2^ and χ^3^ nonlinearities^29,66,67^. With the silicon-based platform that leverages the cost-effectiveness and excellent compatibility of the mature CMOS foundry, only 17 comb lines were recorded near 770 nm via second-harmonic and sum-frequency generations in a microresonator based on a symmetry breaking Si_3_N_4_ platform^66^.

**Fig. 5:**
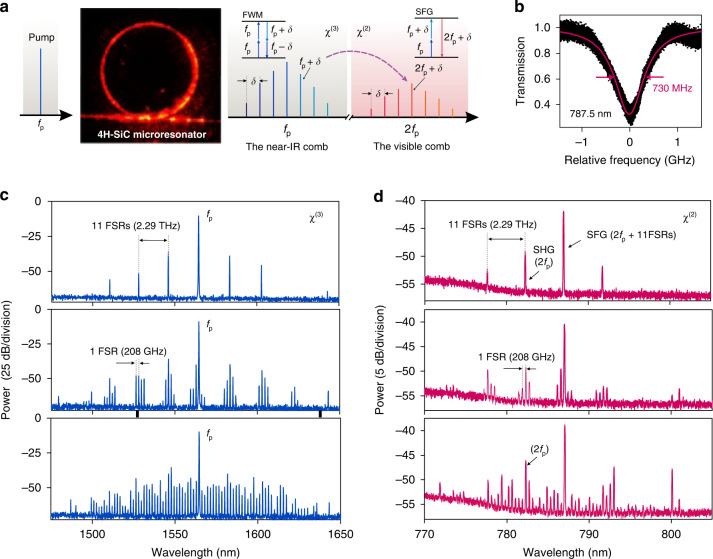
Comb spectral translation from near-IR to visible. **a** Schematic of the comb spectral translation through second- and third-order nonlinearities in the 4H-SiC microresonator. FWM four-wave mixing, SFG sum-frequency generation. The optical image shows the frequency-doubled pump scattered from the microresonator. **b** A resonance profile at 787.5 nm with a Lorentzian fit showing a linewidth of 730 MHz, corresponding to loaded *Q* factor of 5.26 × 10^5^. **c**, **d** Optical spectra of the near-IR and visible combs with different frequency detuning

Compared with the Si_3_N_4_ platform, SiC is particularly attractive as it exhibits both significant χ^2^ and χ^3^ nonlinearities. Here, we demonstrate the generation of broadband visible microcombs in a 4H-SiC microresonator. The experimental setup in this part is similar to the soliton formation experiment, except that the auxiliary laser is not used. The excited signal is collected by the tapered fiber and sent to two OSAs with different target bands. Upon increasing the pump laser power and tuning into a high *Q* resonance, the translated red light strongly scattered from the device is captured by a visible camera CCD, as shown in Fig. 5(a). The red-light comb is generated through FWM and the sum-frequency process. A near-IR pump laser at frequency *f*_*p*_ generates a microcomb centered around the pump in the SiC microresonator through χ^3^-based FWM. Simultaneously, when the power of near-IR comb lines is sufficient, the visible comb around frequency 2*f*_*p*_ is generated via the χ^2^-based SHG and SFG processes. Both near-IR and visible comb lines have the same spectral spacing *δ* (FSR). An efficient χ^2^-based process requires phase-matching between the infrared and visible mode involved in the nonlinear process, and a high *Q* factor resonance is also necessary. As plotted in Fig. 5b, the measured full-width at half-maximum linewidth of a fundamental TM resonance in the visible band is 730 MHz, corresponding to a loaded *Q* factor of 5.26 × 10^5^, about one order of magnitude lower than that in the near-IR band. The degradation may be due to a higher defect absorption loss of SiC at visible wavelength^68,69^. Under the investigated excitation wavelength of 720 nm, the luminescence spectra of various kinds of residual crystal defects such as silicon vacancies and divacancy are observed (see Supplementary Note S5). Although these defects lead to optical absorption in the visible region, as optically active defects, they possess desirable spin coherent properties and hold great promise in quantum technology^37,42,43,70^. The phase matching condition in the current microdisk resonator is not intentionally designed. The corresponding comb spectra in the near-IR and visible are depicted in Fig. 5c, d. When primary comb lines occur in the near-IR, four comb lines are generated in the visible with the same 11-FSR 230 spacing (2.29 THz). Tuning the pump laser further into resonance generates more comb lines that fill the gaps between the primary comb lines, and finally yield single-FSR-spacing combs in both near-IR and visible. The near-IR combs displayed in Fig. 5c are attenuated by 25 dB to protect the OSA. The conversion efficiency from the continuous wave pump light to near-IR combs is *η*_*NIR*_ = *P*_*NIR*_*/P*_*Pump*_ = 2.1%, as expected for Kerr comb generation in the anomalous GVD regimes. Here, *P*_*NIR*_ is the integrated power of near-IR comb lines and *P*_*Pump*_ is the pump power. We define the conversion efficiency of the visible comb as *η*_*VIS*_ = *P*_*VIS*_*/P*_*Pump*_ = 3.3 × 10^−4^%, where *P*_*VIS*_ is the total power of the comb lines in the visible band. The achieved efficiency is one order of magnitude larger than the value reported in an AlN microring resonator^67^. It is observed that the SHG comb lines with doubled frequency 2*f*_*p*_ have lower intensity than that of the SFG comb lines, which is ascribed to the lack of precise phase matching. The phase matching requirement for SHG is more stringent than that of SFG. This phenomenon indicates that the conversion efficiency would be further improved through dispersion engineering. Pumping another resonant mode with a power of 150 mW around 1570 nm can obtain more than 150 converted comb lines, which be clearly distinguished in the recorded spectrum (see Supplementary Note S6). It should be noted that the visible combs observed in this work are chaotic, since they are converted from the near-IR combs in the modulation-instability state. The spectral translation of soliton states via second-order nonlinearity is a challenge for the current microdisks due to insufficient power of soliton states and weak fiber-taper coupling. To obtain coherent visible combs, a promising strategy is to use SiC microring resonators, which have advantages in high-power soliton operability and designable efficient waveguide coupling. Nevertheless, the spectral translation capability of SiC observed here shows the potential of SiC soliton microresonators for *f-2f* self-referencing on a chip.

## Discussion

In this article, we have demonstrated soliton formation in the telecommunication band on the 4H-SiCOI platform. We have observed comb spectral translation from near-IR to visible via the second- and third-order nonlinearities of SiC. The SiC integrated photonics platform exhibiting simultaneous Kerr and χ^2^ nonlinearity is also promising for quantum photonics and electro-optics, e.g., to build integrated quantum light source^37^ and high-speed electro-optic modulators^39,71^. In addition, as 4H-SiC is piezoelectric^72^, monolithic 4H-SiC photonic circuits could enable simultaneous photonics and MEMS functions, e.g., piezoelectric control of photonic microresonators^73^. As SiC is already widely used for RF devices^44^, this platform has already proven to be compatible with CMOS foundries. These key features highlight the prospect of the SiC integrated platform for future low-cost, low-loss, hybrid, and integrated photonics. Therefore, the demonstration reported in this work is an essential step toward industry-compatible integrated nonlinear photonics.

## Materials and methods

### 4H-SiCOI fabrication

The surface of the 4-inch 4H-SiC wafer and the oxidized Si substrate were activated by O_2_/N_2_ plasma, then the two wafers were directly bonded at room temperature. In order to enhance the bonding strength, the bonded wafer was annealed at 400 °C. After annealing treatment, the thickness of the bonded SiC layer was reduced from 500 µm to sub 10 µm by the mechanical grinding process. The grinding process involves a diamond-resin bonded wheel to mechanical remove the SiC layer. Then, the wafer was cut into 10 mm × 12 mm dies, and each die was further thinned down to the desired thickness by inductively-coupled-plasma (ICP) reactive ion-etching (RIE) with SF_2_/O_2_ plasma and chemo-mechanical polish (CMP) processes.

### Fabrication of high-Q microresonator

Microresonators were fabricated from a 4H-SiCOI die with SiC thickness of 800 nm and a buried SiO_2_ thickness of 2 µm. Femtosecond laser micromachining was used to directly pattern the SiC layer. The 800 nm femtosecond laser generated from a Ti: sapphire setup (Libra, Coherent, Inc.) was focused into an ~1 µm diameter focal spot using an objective lens (Nikon LU Plan, 100×/NA 0.7), and the micromachining was carried out at a scan speed of 10 mm s^−1^ of the focused laser spot. The sidewall defined by the femtosecond laser is rough. Next, the CMP process was performed to smooth the sidewall of the 4H-SiC microdisk using a wafer lapping polishing machine. Lastly, to form a suspended microresonator, a diluted HF solution was used to partially remove the SiO_2_ layer beneath the SiC layer.

### Soliton microcomb characterization

For the soliton formation experiment, a tapered fiber was used to couple the pump and auxiliary lasers into and out from the microresonator. The pump and auxiliary lasers from two tunable external cavity diode lasers (DLC CTL 1550, TOPTICA Photonics Inc.) were amplified with two erbium-doped fiber amplifiers (EDFA, Beijing Keyang Optoelectronic Technology Co., Ltd.) and delivered to the microresonators in opposite directions by two circulators. The fiber polarization controllers (FPCs) were used to control the polarization of each laser. The soliton microcomb was initiated by controlling the auxiliary laser to be at the blue-detuned position of one resonance while scanning the pump laser across another resonance. The scan speed of the pump laser of 0.225 GHz ms^−1^ was controlled by an arbitrary function generator (AFG3052C Tektronix Inc.). The generated signal was collected through the same tapered fiber, and its spectral was sent into an optical spectrum analyzer (OSA: AQ6370D, YOKOGAWA Inc.) for analysis. One part of the generated laser through a coarse wavelength division multiplexer (CWDM) with the bandwidth of 20 nm was used to filter out several comb lines to record the power trace during the scan process. The residual part was sent to the dense wavelength division multiplexer to filter out two adjacent comb lines to beat note via the method of cascading the intensity modulator (IM) and the phase modulator (PM)^74^. The RF beat note signal was recorded by the electrical spectrum analyzer (ESA ROHDE & SCHWARZ Inc.). The repetition rate is calculated as *NΩ* ± *f*_*b*_, where *N* = 15 is the order of the generated sidebands, *Ω* = 13.865 GHz is the RF signal frequency, *f*_*b*_ is the RF signal frequency. Given the measured RF beatnote as depicted in the inset of Fig. 3d, the repetition rate can be calculated as 208.12 GHz, which is agreed well with the size of the SiC microdisk with a radius of 98 µm.

### Soliton microcomb spectral fitting

For the single soliton, the optical field has the form in time domain^2^

1 $$E\left( t \right)\sim {{{\mathrm{sech}}}}\left(\frac{t}{{T_0}}\right) \otimes \mathop {\sum}\limits_{n = - \infty }^\infty {\delta (t - nT)}$$

where t and *T*_0_ are the time and the width of a soliton pulse, respectively. *T* represents the pulse period and equals to microcomb line spacing *f*_*m*_. Transforming Eq. (1) to the frequency domain by Fourier Transform, the spectrum of the single soliton microcomb can be written

2 $$\tilde E\left( f \right)\sim {{{\mathrm{sech}}}}(\pi ^2T_0f)\mathop {\sum}\limits_{k = - \infty }^\infty {\delta (f - kf_m)}$$

where $k \in Z$ is the mode index of with respect to the pump line.

In the similar manner, two solitons microcomb can be given

3 $$E\left( t \right)\sim \left[{{{\mathrm{sech}}}}\left( {\frac{t}{{T_0}}} \right) + {{{\mathrm{sech}}}}\left( {\frac{{t - \frac{{\alpha T}}{{2\pi }}}}{{T_0}}} \right)\right] \otimes \mathop {\sum}\limits_{n = - \infty }^\infty {\delta (t - nT)}$$

4 $$\tilde E\left( f \right)\sim {{{\mathrm{sech}}}}(\pi ^2T_0f)(1 + e^{ - i\alpha k})\mathop {\sum}\limits_{k = - \infty }^\infty {\delta (f - kf_m)}$$

where $\alpha \in [0,2\pi ]$ is introduced to represent the angle of these two pulses along the cavity round trip.

Equations (2) and (4) were used to fit the experimental spectrum. Firstly, the experimental spectrum was detected and marked with their relative mode index from the pump mode. Note that the pump mode is rejected due to it is sharp peak. Then, on the known parameter *f*_*m*_, the set of fitting parameters [*α*, *T*_*0*_] are estimated to best fit the experimental spectrum accordingly to Eqs. (2) and (4). The simulation results match well with the experimental data.

## Supplementary information

Supplementary Materials for Soliton Formation and Spectral Translation into near-Visible on CMOS-Compatible 4H-Silicon-Carbide-on-Insulator Platform

## Data Availability

The data that support the plots within this manuscript and other findings of this study are available on Zenodo (10.5281/zenodo.6510121). All other data used in this study are available from the corresponding authors upon reasonable request.
