# Supplementary material for: Soliton formation and spectral translation into visible on CMOS-compatible 4H-silicon-carbide-on-insulator platform
Source: Light Sci Appl. 2022 Dec 7;11:341. doi: 10.1038/s41377-022-01042-w (PMC9726892; doi:10.1038/s41377-022-01042-w)
Supplement: Supplementary file 1 — Supplementary Materials for Soliton Formation and Spectral Translation into near-Visible on CMOS-Compatible 4H-Silicon-Carbide-on-Insulator Platform [file 41377_2022_1042_MOESM1_ESM.docx]

Supplementary Materials for

**Soliton Formation and Spectral Translation into near-Visible on CMOS-Compatible 4H-Silicon-Carbide-on-Insulator Platform**

Chengli Wang^†^, Jin Li^†^, Ailun Yi1^†^, Zhiwei Fang^†^, Liping Zhou, Zhe Wang, Rui Niu , Yang Chen , Jiaxiang Zhang , Ya Cheng , Junqiu Liu^∗^, Chun-Hua Dong^∗^, and Xin Ou^∗^

∗ Corresponding authors: liujq@iqasz.cn, chunhua@ustc.edu.cn, ouxin@mail.sim.ac.cn.

**This PDF file includes:**

Supplementary Text

Figs. S1 to S6

References (1 to 8)

Supplementary Note S1. Coupling states

The resonators were coupled through a tapered optical fiber with a diameter of about 1 μm. A piezo-electrical transducer (PZT) was used to control the coupling gap between the microresonator and the tapered fiber. Therefore, the under, critical, and over coupling states can be controlled. The top, middle, and bottom of Fig. S1 show the measured transmission traces for these three coupling states, respectively. The inset of each figure schematically shows the relative position of the microdisk and the optical fiber.

Mapping the transmission spectra as a function of the gap between the fiber and the microresonator helps identify the fundamental mode families. In the transverse-magnetic polarization, a mode family labeled by yellow shapes is distinctly observed in under coupling states (the top of Fig.S1), with an equally free spectral range (FSR) of about 1.72 nm. The fundamental TM mode families will appear first in the under-coupling states, we identified the labeled mode family as fundamental TM mode. This FSR value is consistent with the finite-element simulation results.

When the taper is placed at a precise distance away from the microdisk, the critical coupling is achieved, resulting in minimum transmitted power and maximum in-cavity power in the resonances, as shown in the middle of Fig.S1. When the optical fiber is in direct contact with the microdisk, a large number of modes are excited in the over-coupling state (the bottom of Fig.S1). However, the relative position of the marked mode family is unchanged in different coupling states, which helps us to distinguish the fundamental TM mode among a large number of mode families.


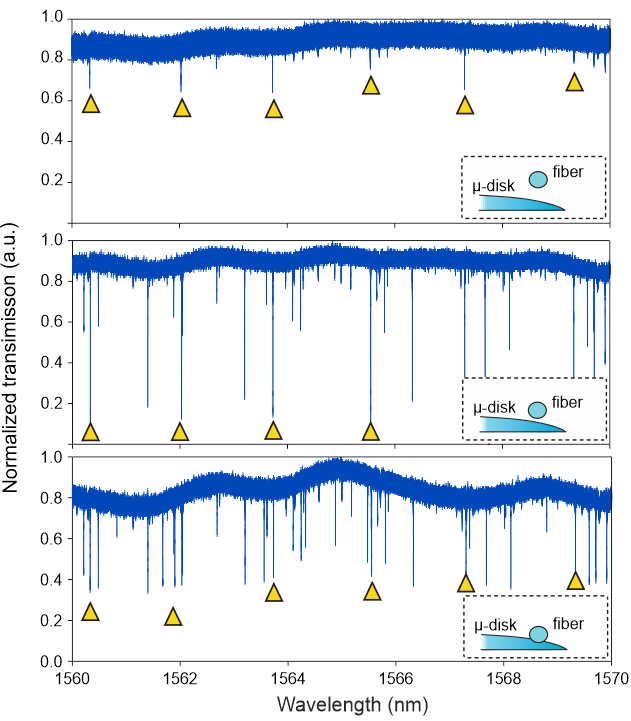


FigS1. The over, critical, and under coupling states can be controllably achieved by adjusting the gap between the fiber and the microresonator. The inset of each figure schematically shows the relative position of the microdisk and the optical fiber.

Supplementary Note S2. Optomechanical effect in critical coupling states.

Although maximum circulating power can be achieved in critical coupling states, the coupling efficiency is sensitive to any displacement fluctuations due to acoustic and mechanical noise^1^. Moreover, due to the small volume and high *Q* factor, the microresonator is also highly susceptible to nonlinear effects of its own, such as optomechanical effects^2^.

In the critical coupling state, as shown in Fig.S2(a), we observe optomechanical oscillation via radiation-pressure in the triangular-shaped pump transmission when the pump power is more than 30 mW. The detail of the oscillation displayed in the inset shows beat frequency characteristics. To measure the frequency characteristics, a high-speed photodetector with a bandwidth of 300 MHz is used to detect the transmission signal, and the detected signal is then sent to an RF spectrum analyzer. Fig.S2(b) shows the measured RF spectrum, we can clearly observe a series of peaks, which originate from mechanical modes in the microresonator. Three marked mechanical resonances can be visualized with the FEM simulation as shown in the inset of Fig.S2(b). For these three modes, the simulated eigen-frequencies are in good agreement with the measured frequencies (15.3 MHz, 53.2 MHz, and 67.5 MHz). The fundamental radial breathing mode (RBM) exhibits the generation of a harmonic family up to sixth order (limited by the performance of the photodetector), implying a strong optomechanical gain in our cases.


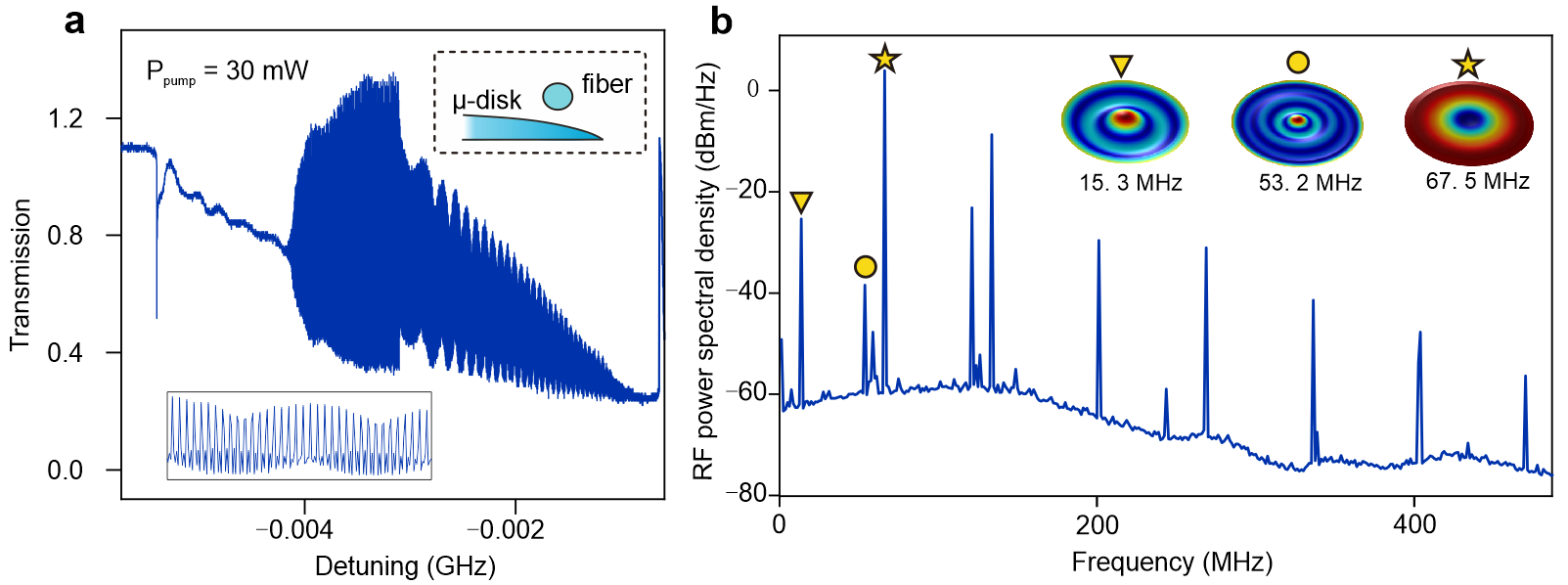


FigS2. (a) Transmitted optical power is measured as the pump is scanned into an optical resonance. The oscillation is observed at a certain detuning position. The top right inset schematically shows the critical coupling state. The bottom left inset enlarges the oscillation spectrum. (b) RF spectrum of the oscillation transmission. The FEM simulated displacement amplitude profiles of the marked mechanical resonances are also shown.

Supplementary Note S3. Nonlinear refractive measurement.

Soliton comb was initially realized through an optical parametric oscillation (OPO) process. The onset of the typically measured OPO spectra is shown in Fig. 1(e). It shows that a series of widely spaced sidebands were generated at the input power of around 13 mW. The output power of the primary sidebands as a function of the input power was plotted in Fig.S3, revealing a threshold of 12.9 mW. This value provides us to evaluate the $\chi^{3}$ nonlinearity of 4H-SiC by the expression

$$P_{th}\approx1.54(\frac{\pi}{2})\frac{Q_{c}}{{2Q_{L}^{3}}}\frac{n^{2}LA_{eff}}{{\lambda n}_{2}}$$

where $n=2.6$ is the refractive index, $\lambda$ is the pump wavelength, the effective mode area $A_{eff} \approx3.2 {\mu m}^{2}$ can be calculated using optical mode solver (Lumerical Mode Solution). The coupling quality factor $Q_{c}$ and the loaded quality factor $Q_{L}$ are extracted from the resonance. Given the measured $P_{th}=12.9 mW$, the nonlinear refractive index can be inferred as $n_{2}=6 \times{10}^{-19} m^{2}/W$. This value is consistent with previously reported results^3^.


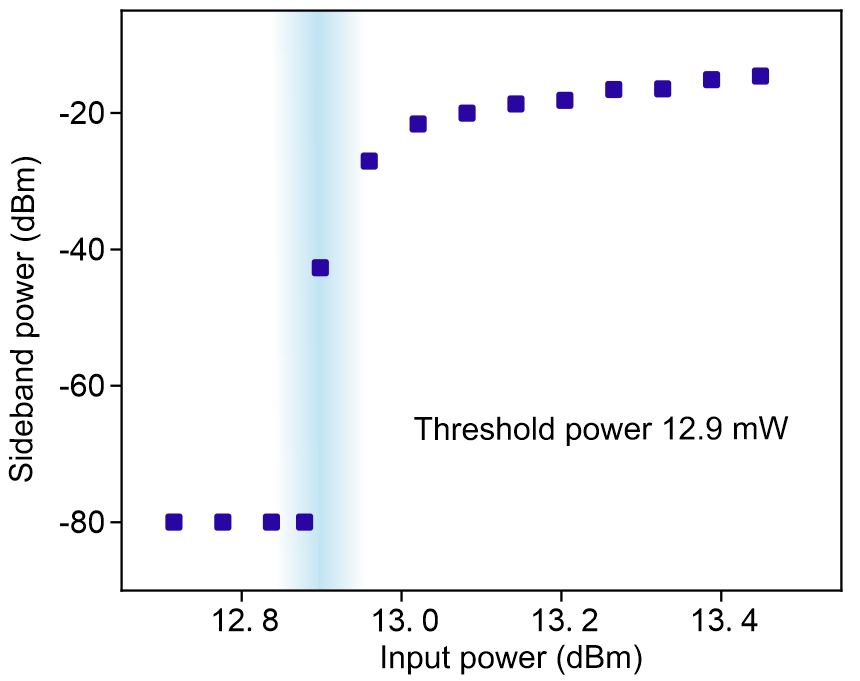


Fig.S3 The primary sideband power as a function of the pump power, revealing a threshold of 12.9 mW for a SiC microresonator.

Supplementary Note S4. Thermal-optic coefficient of the 4H-SiC microdisk resonator.


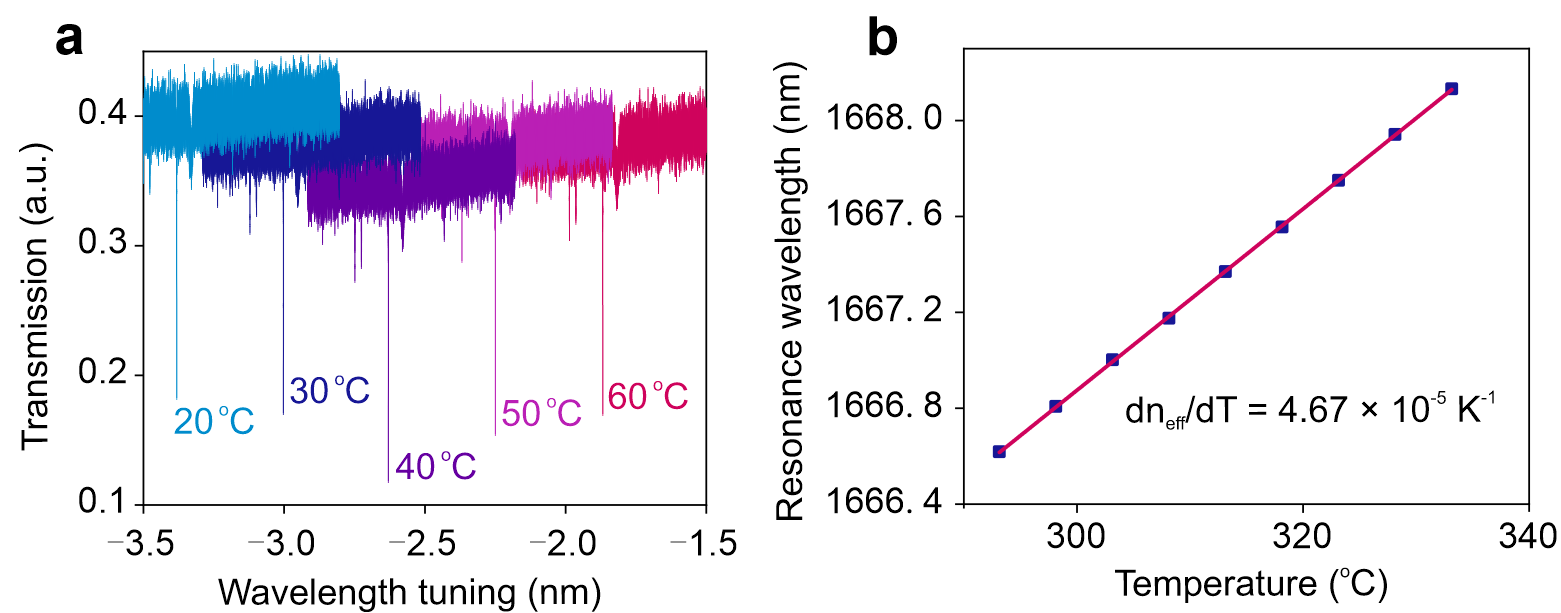


Fig. S4 (a) Transmission vs. wavelength for a resonance in the microdisk at different temperatures. (b) The wavelength of resonance as a function of the temperature, with experimental data shown in dots and linear fitting in the solid red line.

Thermo-optic effects in optical resonators play a crucial role during the soliton microcomb formation process. To measure the thermo-optic coefficient of the fabricated 4H-SiC resonator, we changed the chip temperature from 293.15 K to 333.15 K (20℃ to 60℃) and recorded the resonance transmission. Fig. S4(a) shows the recorded resonance transmission spectrum with different temperatures. The cavity resonances shift as a function of the temperatures is plotted in Fig. S4(b). The temperature(*T*)-dependent resonance wavelength *λ* is mainly determined to both thermo-optic and thermal expansion effects, which is given by

$\frac{1}{\lambda}\frac{d\lambda}{dT}= \frac{1}{n_{eff}}\frac{dn_{eff}}{dT}+ \frac{1}{R}\frac{dR}{dT}$ (1)

where $n_{eff}$ is the effective refractive index of the resonant mode, *R* is the radius of the microresonator. In the investigated temperature range, the cavity resonances change linearly with the temperature, with a fitting slope of *dλ/dT* = 0.03782 ± 0.0001 nm K^-1^. The temperature-dependent of the radius is obtained by the linear thermal expansion coefficient of 4H-SiC, with a value of *(1/R)(dR/dT)* = 3.21 × 10^-6^ K^-1^ at room temperature^4^. Accord toing the Eq. (1), we can obtain the thermo-optic coefficient *d*$n_{eff}$*/dT* = 4.67 × 10^-5^ K^-1^ at room temperature.

**Supplementary Note S5. The photoluminescence spectrum under the visible laser excitation.**

**
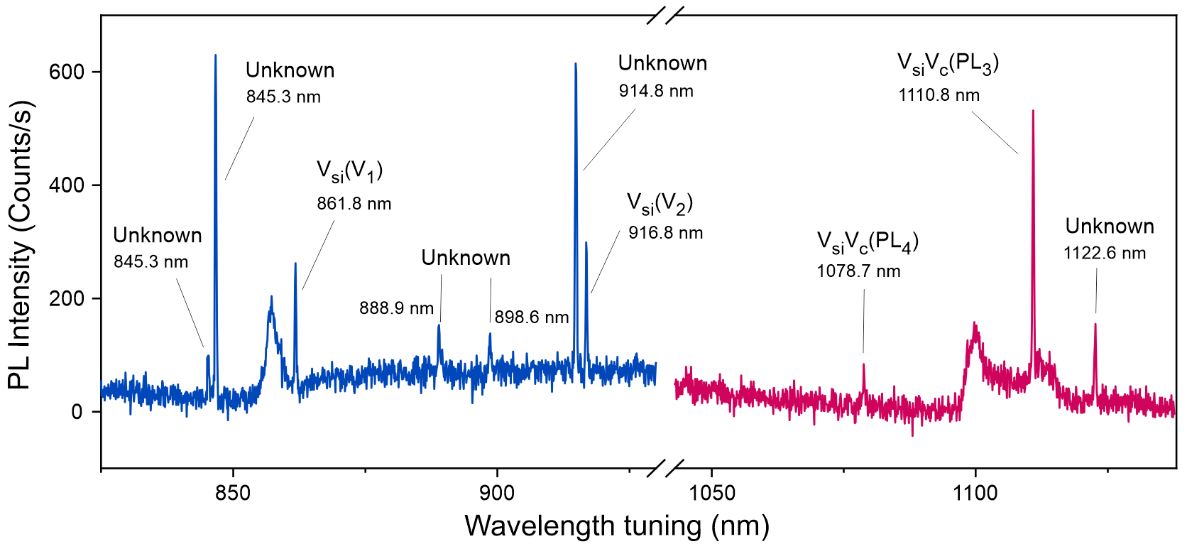
**

Fig. S5 The photoluminescence spectrum. The signal collected at T = 8K using a home-built setup. Zero phonon lines associated with various kinds of defect are labeled. Several lines label “Unknown” mean the origin of these lines are currently unknown.

A photoluminescence spectrum of the 4H-SiCOI sample is shown in Fig.S5. These data are collected by employing a cryogenic micro-photoluminescence setup. The 4H-SiCOI sample is placed inside a liquid helium cryostat and cooled down to 8K. A continuous wave 720 nm laser with a power up 1 mW is pumped and reflected off by a dichroic mirror toward an objective, and finally addressed the sample. The emitted PL signal is collected by the same objective, then transmitted through the dichroic mirror and collected though a single mode fiber. Filtered signal is delivered to a high-resolution spectrometer equipped with a thermoelectric-cooled Si charge-coupled device. A series of sharp lines are apparent, along with several of broader features. These sharp lines named zero phonon lines originate from the residual optical active defects, labeled in the spectrum. According to the zero phonon lines of various defects reported in previous literatures, we can distinguish that the emission at wavelengths of 861.8 nm and 916.8 nm assigned to the silicon vacancy (V_si_)^5^. V1 and V2 originate from the inequivalent lattice sites for the V_si_ in 4H-SiC. Similarly, emission lines at the wavelengths of 1078.7 nm and 1110.8 nm can be identified to originate from two distinct forms of the natural divacancy (V_si_V_C_)^6^. The other sharp zero phonon lines marked “Unknown” suggests their origins are currently unidentified^7,8^.

These result reveals that the 4H-SiCOI host for naturally occurring color centers. Although these defects lead to optical absorption in visible region, their desirable spin coherent properties and hold great promise in quantum technology.

Supplementary Note S6. The optical spectrum of a broadband spectral translation from near-IR combs to visible-comb.

In this work, both the near-IR [Fig.S6(a)] and visible comb [Fig.S6(b)] spectra are recorded by two separated optical spectrum analyzers (OSA, Yokogawa AQ6370D, 600 nm to 1700 nm). Due to the relatively high background noise of the OSA near the visible band, the counted visible comb lines are limited by the instrument sensitivity. By pumping a resonant mode near 1572.2 nm with a power of 150 mW, more than 150 converted comb lines can be distinguished in the visible spectrum, as depicted in Fig.S6(b). The inset figure in Fig.S6(b) enlarge the counted comb lines.


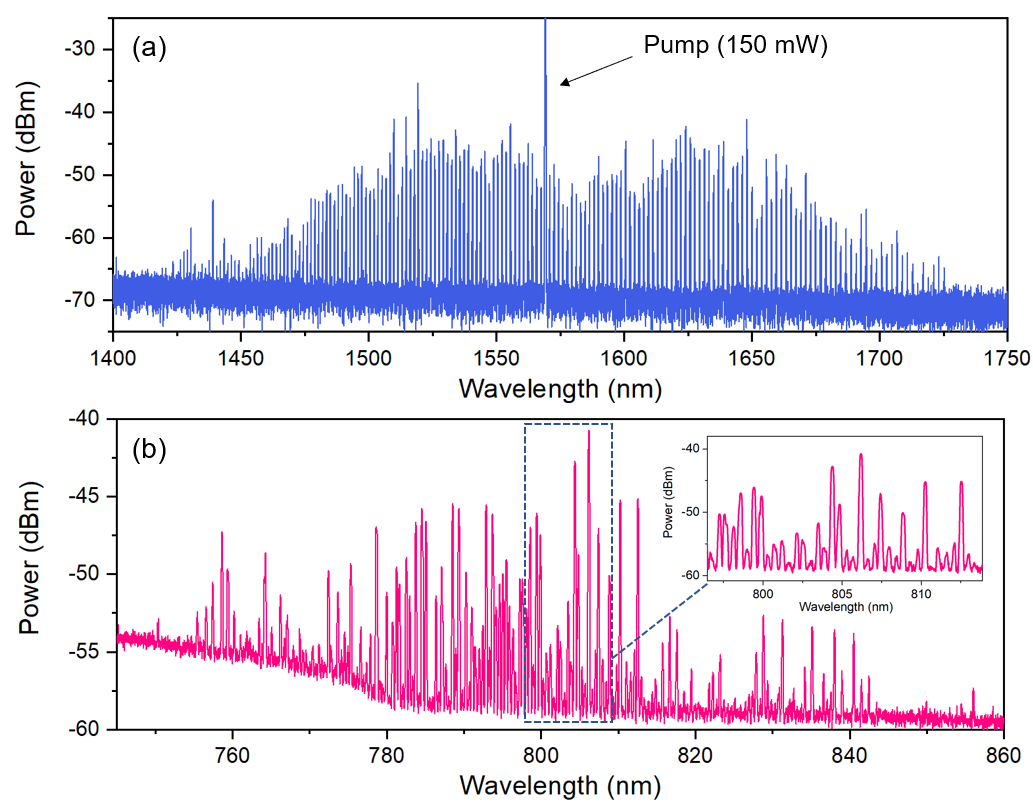


Fig. S6 The complete optical spectrum of near-IR (a) and visible (b) frequency microcombs. The power mismatch of the red and blue spectrum is originated from the spectrograph efficiency variation over the different spectral ranges. 150 mW pump power is sent into the cavity via a tapered fiber. More than 150 comb lines with a coverage of about 100 nm and a spacing of 208 GHz can be distinguished in near-visible wavelength.

References

1 Chow, J. H. *et al.* Critical coupling control of a microresonator by laser amplitude modulation. *Opt. Express* **20**, 12622-12630 (2012).

2 Aspelmeyer, M., Kippenberg, T. J. & Marquardt, F. Cavity optomechanics. *Reviews of Modern Physics* **86**, 1391 (2014).

3 Guidry, M. A. *et al.* Optical parametric oscillation in silicon carbide nanophotonics. *Optica* **7**, 1139-1142 (2020).

4 Li, Z. & Bradt, R. C. Thermal expansion of the hexagonal (4 H) polytype of SiC. *J. Appl. Phys.* **60**, 612-614 (1986).

5 Nagy, R. *et al.* High-fidelity spin and optical control of single silicon-vacancy centres in silicon carbide. *Nat. Commun.* **10**, 1-8 (2019).

6 Christle, D. J. *et al.* Isolated electron spins in silicon carbide with millisecond coherence times. *Nat. Mater.* **14**, 160-163 (2015).

7 Riedel, D. *et al.* Resonant addressing and manipulation of silicon vacancy qubits in silicon carbide. *Phys. Rev. Lett.* **109**, 226402 (2012).

8 Koehl, W. F., Buckley, B. B., Heremans, F. J., Calusine, G. & Awschalom, D. D. Room temperature coherent control of defect spin qubits in silicon carbide. *Nature* **479**, 84-87 (2011).
